# Silver Therapy

**Published:** 1906-12

**Authors:** 


### ABSTRACTS.

### SILVER THERAPY.

Solomon Solis Cohen, Philadelphia, writing on the subject, (*Saint Louis Med. Rev.,* February 24, 1906) says that the knowledge of the power of colloidal silver (collargol) in septic and septico-toxic conditions should be made more general. He has seen two recoveries from malignant endocarditis and numerous recoveries from other grave infections by its energetic employment intravenously and per rectum. Suppositories may be used instead of rectal injections, and they are especially available in children. An adult may be given 2 to 3 gr., a child 1 gr., in each suppository. Although the solution is itself antiseptic, no precaution should be neglected when intravenous injection is done. It is best to use an all-glass syringe with a platino-iridium needle and with a tight but smooth conical metal attachment between needle and syringe. The needle may be inserted into a superficial vein of the hand, forearm, or flexure of the elbow after the vein has been made prominent by pressure above. When blood issues from the needle, the syringe is attached, pressure relaxed, and injection made. Fifteen to 60 min. of a 2% to 5% solution, made with sterilized, distilled water, should be used. One intravenous or two rectal injections daily usually suffice. As improvement follows, the intervals between injections may be lengthened.

Several cases observed recently at the Philadelphia Hospital are worthy of mention. Used promptly after chill, in the stage of congestion, as evidenced by dulness and crepitant rales, colloidal silver seemed to abort an attack of pneumonia. At all events the temperature fell within 48 hours from 104°F. to normal, sweating occurred, respiration fell from 40 to 28, pulse from 120 to 90; and within 3 days large moist rales were present in the dull area, which cleared up completely in about 7 days from the first appearance of symptoms. In several cases of broncho-pneumonia complicating typhoid fever, improvement rapidly followed the institution of the treatment. In one case of tuberculous diarrhea, with tubercle bacilli in the feces but no visible lung signs, the rectal injection of colloidal silver quickly controlled the symptoms. The patient left the hospital apparently well with feces seemingly free from tubercle bacilli; at least, repeated examinations by Dr. Rosenberger, director of the pathological laboratory, failed to show any. Cohen has been less successful in controlling the mixed infection of phthisis by colloidal silver. German physicians advocate its use twice daily by mouth and once daily by rectum. The dose is about 2 grains each time. In a case of streptothrix phthisis, however, great improvement in general condition and in pulmonary signs, with lessening of the quantity of septum and apparent attenuation of the streptothrix culture, has followed the use of colloidal silver. In a case of bronchopneumonia in an infant with severe meningeal symptoms, a recovery followed the use of colloidal silver suppositories, aided by the injection of colloidal silver ointment over the site of the cervical blister.

The usual fault in the administration of this agent is delay and timidity. It must be used promptly and freely, and when so used the results are gratifying. The indications are septicemia and the toxemia of infection, without regard to the special microorganism concerned. The author has not seen from inunction results at all comparable to those obtained by intravenous or rectal injection.

